# THE ASSOCIATION BETWEEN DIGITAL ENGAGEMENT OF OLDER ADULTS AND DISPLACEMENT DURING ARMED-CONFLICT CIRCUMSTANCES

**DOI:** 10.1093/geroni/igae098.1654

**Published:** 2024-12-31

**Authors:** Ittay Mannheim, Rinat Lifshitz, Yaacov Bachner, Ella Cohn-Schwartz

**Affiliations:** Ben Gurion University of the Negev, Beer-Sheva, HaDarom, Israel; The Max Stern Academic College of Emek Yezreel, Tel Adashim, HaZafon, Israel; Ben Gurion University of the Negev, Beer-Sheva, HaDarom, Israel; Ben Gurion University of the Negev, Beer-Sheva, HaDarom, Israel

## Abstract

Digital engagement is perceived as having the potential to improve the well-being of older adults. This study tests this notion in extreme circumstances, such as an ongoing military conflict. The association of digital engagement with the well-being of older adults was examined within the context of forced displacement and imminent threat (e.g., rocket attacks) due to the ongoing war in Israel. Ninety-three older persons that were evacuated from their homes, and 150 that were not evacuated responded to an online or paper survey. The survey measured their digital engagement before the war and in the weeks after it began, perceptions towards aging and technology, and well-being (depressive symptoms, loneliness, subjective health, and control). Evacuees were found to be lonelier, with lower subjective health and a lower sense of control compared to non-evacuees. Baseline digital engagement retrospectively reported from before the war was the same between groups. However, following the war, those evacuated reported using a lower number of devices, had different patterns of uses, and perceived higher limitations in using technology. Importantly, a stepwise regression analysis identified that digital engagement was a stronger predictor than displacement on higher loneliness and lower subjective health. Whereas rocket threat was the strongest predictor for depressive symptoms and displacement for sense of control. Negative perceptions towards aging and technology were also predictors of higher depressive symptoms. This study discusses the importance of digital engagement for the well-being of the older population in a world with ever-growing prevalence of extreme circumstances such as armed conflicts.

